# Determination of carbon in microplastics and single cells by total consumption microdroplet ICP-TOFMS

**DOI:** 10.1007/s00216-023-05064-0

**Published:** 2023-12-08

**Authors:** Thomas Vonderach, Alexander Gundlach-Graham, Detlef Günther

**Affiliations:** 1 Department of Chemistry and Applied Biosciences, ETH Zurich, Vladimir Prelog Weg 1, 8093 Zurich, Switzerland; 2 Department of Chemistry, Iowa State University, 2415 Osborn Drive, 1605 Gilman Hall, Ames, IA 50011-1021 USA

**Keywords:** Cell systems, Single-cell analysis, Mass spectrometry, ICP-MS, Nanoparticles, Nanotechnology

## Abstract

Recently, spICP-MS analyses of microplastics have demonstrated that the detection capabilities of ICP-MS are sufficient to determine the size and composition of such materials. However, solution nebulization or microdroplet generation limits the sizes of droplets, microparticles, or cells that can be efficiently introduced into commonly used horizontal ICP-MS configurations. Therefore, we introduced the development of a downward-pointing ICP coupled to a time-of-flight mass spectrometer (ICP-TOFMS), which enables quantitative transport of large microdroplets (diameters up to 90 µm) into the ICP. Here, we report the capabilities of downward ICP-TOFMS for the quantitative analysis of single cells and microplastic particles. For calibration of element mass amount per particle or cell, microdroplets (70 µm diameter) composed of multielement solutions were measured by ICP-TOFMS. Microplastic beads (polystyrene) and spleenocyte cells were then also embedded in microdroplets and measured by ICP-TOFMS with ion optics optimized to determine the signals from ^12^C^+^ and other isotopes of interest, including ^140^Ce, ^153^Eu, ^165^Ho, and ^175^Lu from the REE beads and ^31^P for the cells. The results achieved using the prototype instrument of a vertical downward-pointing ICP-TOFMS demonstrate that such a plasma configuration is well suited to analyze microplastics and single cells. For single microbead and cell analyses, the critical mass for carbon was 4.8 pg, and the mean determined carbon mass amounts were 14 and 23 pg, respectively. For the microbead analysis, the detected carbon mass corresponds to a particle diameter of 2.93 ± 0.24 µm, which is consistent with the scanning (transmission) electron microscopy–determined diameter of 2.97 ± 0.04 µm. Furthermore, in the analysis of spleenocyte cells, carbon and phosphorus masses were determined to be correlated.

**Graphical abstract:**

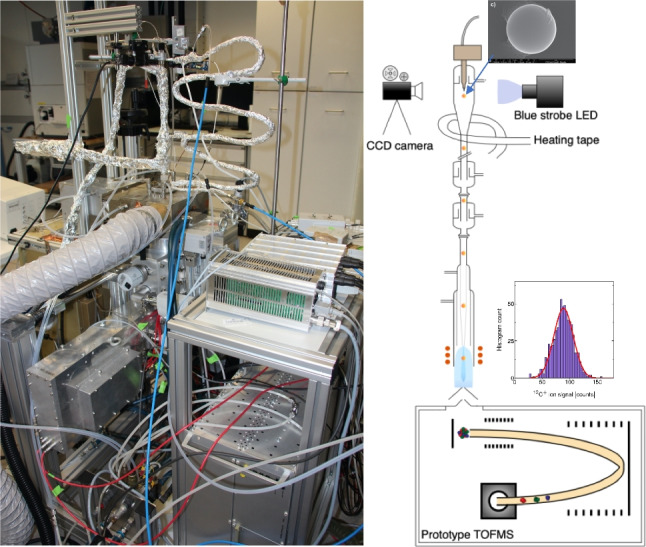

**Supplementary Information:**

The online version contains supplementary material available at 10.1007/s00216-023-05064-0.

## Introduction

Biological cells contain up to 70 wt% water, but also contain inorganic salts and organic substances such as carbohydrates, lipids, proteins, and nucleic acids [1]. Carbon (C) is present in these cell-specific organic compounds, which make up most of the dry weight of cells [2]. The accurate quantification of carbon at single-cell level would allow a more precise determination of cellular biomass, which could reveal differences between populations or cell lines. This might be, for example, useful in cell-line selection processes such as those found in therapeutic protein production. In such processes, mammalian cells (e.g., CHO cells) are commonly used and engineered toward fast growth and protein production rates [3, 4].

Single-cell inductively coupled plasma–mass spectrometry (scICP-MS) [5] is an emerging area of research that enables the direct investigation of individual and (in the best case) intact cells to quantify either endogenous elements, dissolved metal or nanoparticle uptake amounts [6–8], or metal labels for biomolecule detection [9, 10], as in mass cytometry [11–13]. Previously, scICP-MS has been used to measure the endogenous content of magnesium, phosphorus, calcium, manganese, iron, copper, zinc, and selenium in bacterial [14–17] and mammalian cells [18]. However, the measurement of carbon content in these cells is also possible via ICP-MS and, when combined with the analysis of other endogenous elements, could provide new insights about cell-to-cell heterogeneity. While the quantification of carbon in single cells by ICP-MS is not common practice, the analysis of carbon as an internal standard for laser ablation ICP-MS has been studied [19]. In addition, recent work in single-particle (sp) ICP-MS has demonstrated the ability to measure and quantify carbon (either using ^12^C or ^13^C) for the determination and analysis of microplastic particles [20–22]. The measurement of microplastics in environmental samples is a pressing analytical challenge due to the potential environmental impact and lack of suitable high-sensitivity, high-throughput analytical approaches [23–25]. For example, Gonzalez de Vega and co-workers [26] reported on the determination of the carbon content in microplastics and different algae species in a seawater matrix using single-particle/cell ICP-MS and ICP-MS/MS.

For the measurement and quantification of the carbon content of individual cells, sample preparation remains a crucial challenge; single-cell analyses are usually highly cell type–specific and depend on the underlying problem or question. For the quantification of carbon, critical issues involve washing steps that could lead to a washout of proteins, carbohydrates, lipids, or amino acids. Even a simple procedure, such as the resuspension of cells in water for scICP-MS analysis, can cause osmotic stress (or lysis) and thus the release of cytosolic species or even cell compartments. Washing solutions containing 0.9% NaCl buffers are well-suited as they are isotonic. Cell fixation might further preserve the cell and its interior, although it might introduce some additional carbon to the cell as typical fixatives such as formalin are C-based compounds. In our work here, we aim to demonstrate the opportunity to measure the carbon content and other endogenous elements in single cells. The eventual applicability of the approach will likely be on a case-by-case basis and require more extensive validation.

Another challenge in measuring and quantifying carbon content in microplastics or cells is the elevated C^+^ background in ICP-MS, which originates from ambient atmospheric CO_2_, dissolved CO_2_ in sample solutions, and any other carbon traces in the plasma/carrier gases (Ar and He) used [27]. Several studies have reported different approaches to reduce such background. Notably, Vogl and Heumann [28] were able to reduce the carbon background by a factor of three by acidifying the solution followed by purging with He. Even in cases in which the ICP background cannot be reduced, the addition of O_2_ as reaction gas using an ICP-MS/MS system has been used to convert ^12^C^+^ into ^12^C^16^O^+^ which was suggested for the analysis of seawater and algae cells [26]. Bolea-Fernandez and co-workers [20] investigated polystyrene microspheres by monitoring the ^13^C^+^ signals, while a low sample flow rate was chosen to optimize the signal-to-background ratio as well as the transport efficiency, which was determined to be approximately 50%. Recent studies by Harycki and Gundlach-Graham [22] demonstrated the detection and quantification of carbon in polystyrene bead microparticles of approximately 3 µm by means of online microdroplet calibration. Hendriks and Mitrano [29] reported on a study where carbon in microplastics and algae cells by sp-ICP-TOFMS.

In this study, polymer microbeads and spleenocytes were transported via monodisperse microdroplets into a downward-pointing ICP and further analyzed by inductively coupled plasma–time-of-flight mass spectrometry (ICP-TOFMS) [30]. This total consumption microdroplet-ICP-TOFMS setup allows for multiplexed analysis at high time resolution which enables the precise identification of droplets, beads, and cells according to tracer signals in addition to monitoring their carbon content and other metals present.

## Experimental

### Sample preparation

Since a solution of glucose was suggested as a calibration standard for carbon in earlier studies [27], microdroplets with known size and glucose content were used to externally calibrate for carbon and other elements (e.g., phosphorous and elements contained in the microbeads). A 4000 mg L^−1^ carbon stock solution was prepared from D-glucose (BioXtra, ≥ 99.5% (GC), Sigma-Aldrich) and was further diluted in order to obtain calibration standards containing 50, 100, 150, and 200 mg L^−1^ carbon. A 1 mg L^−1^ Cs stock solution prepared from an ICP standard (Inorganic Ventures, USA) was added to the blank and to the calibration standards for a final concentration of 40 µg L^−1^ of Cs. Cs served as a suitable droplet tracer because it is highly soluble in water and does not require the addition of acid to stabilize the solution. All solutions were prepared in 50 mL centrifuge tubes and were transferred into microdroplet dispenser compatible 4 mL polyethylene (PE) vials (4 mL, Nalgene Natural HDPE, Thermo Scientific, USA) prior to analysis.

2.5 mL of *Four Element Calibration Beads* (EQ4 beads, Fluidigm) was transferred into a 4 mL PE vial and Cs stock solution was added to obtain a final Cs concentration of 50 µg L^−1^ in the bead suspension.

Mouse spleenocytes were received (after harvesting from spleen) as a suspension in PBS (1 mL, approximately 5 × 10^6^ cells). 0.1 mL of cell suspension (approximately 5 × 10^5^ cells) and 0.9 mL ultrapure water were transferred into a 1.5 mL Eppendorf tube, centrifuged, washed two times with 1 mL ultrapure water, and resuspended in 1 mL ultrapure water. The cell suspension was transferred into a 4 mL PE vial and Cs stock solution was added to obtain a final Cs concentration of 100 µg L^−1^.

### Sample introduction system and ICP-TOFMS measurement

The prepared calibration standards and the bead and cell suspensions were fed into a piezo-driven autodrop pipette (microdrop Technologies GmbH, Norderstedt, Germany) to generate monodisperse microdroplets as previously described [31]. Dispensed microdroplets, which all contain Cs as a tracer element and some of which contain analyte microplastic particles or cells, were introduced into a heated helium-filled glass falling tube to accelerate droplet desolvation [32, 33] and then into downward-pointing ICP-TOFMS as previously described [30]. An autodrop pipette with a nominal inner diameter of 70 µm at the dispenser tip was used to generate 70 µm droplets at a frequency of 50 Hz. Even 93 µm droplet sizes were successfully introduced into the plasma; however, 70 µm droplets provided the most stable signals over a longer period of time. A schematic design of the device and the operational parameters are provided in Supporting Information (SI).

The ion optics of the TOF were specifically optimized for maximum carbon sensitivity setting the Einzel lens voltage to 7.4 V, the “skimmer” to 150 V, and the RF amplitude of the notch filter to 1.5 V.

Time-resolved droplet signals were acquired continuously for 2 min per run by recording the mass spectra at a spectral averaging rate of 333 Hz (3 ms average time). For the calibration standards, 1 run per sample, for the beads, 4 runs per sample, and for the cells, 21 runs per sample were conducted. Microdroplet-derived signals were separated from steady-state ICP-MS background using signals from the tracer element, Cs. All elemental signals of interest were then integrated from the found droplet-derived signals and plotted as histograms, fitted by a Gaussian function and background-subtracted using the mean element-specific backgrounds. An external calibration for carbon quantification was carried out by plotting the mean background-subtracted ^12^C^+^ signal values obtained from Gaussian fits of the signal histograms versus the carbon mass per sample droplet, followed by a linear regression. Quantitative carbon data (C content of beads and cells and bead size) are provided as box and whisker plots.

### Sample preparation and instrumentation for S(T)EM

The polystyrene beads were investigated by S(T)EM to obtain information about their shapes and sizes. A few microliters of the bead suspension were deposited onto glow-discharged Si-wafer chips followed by blotting off the excess sample and drying. The mounted sample was then covered with a 20 nm Pt layer using a sputter coating. An ultrahigh resolution SEM (FEI Magellan 400, Thermo Fisher Scientific, Hillsboro, OR, USA) equipped with a Schottky Type FEG was operated at 5 kV (STEM at 30 kV), and secondary electrons were detected via an Everhart–Thornley detector (ETD) or through-the-lens detector (TLD), backscattered electrons via a backscattered detector (CBS) and transmitted electrons via bright field (BF), dark field (DF), or high-angle annular dark field (HAADF). Images from the backscattered electrons as well as from transmitted electrons in HAADF mode were used to measure the particle diameters. Figure 6 illustrates SEM images of polymer beads, for which secondary, backscattered, or transmitted electrons were analyzed.

## Results and discussion

### Time-resolved data

**Fig. 1:**
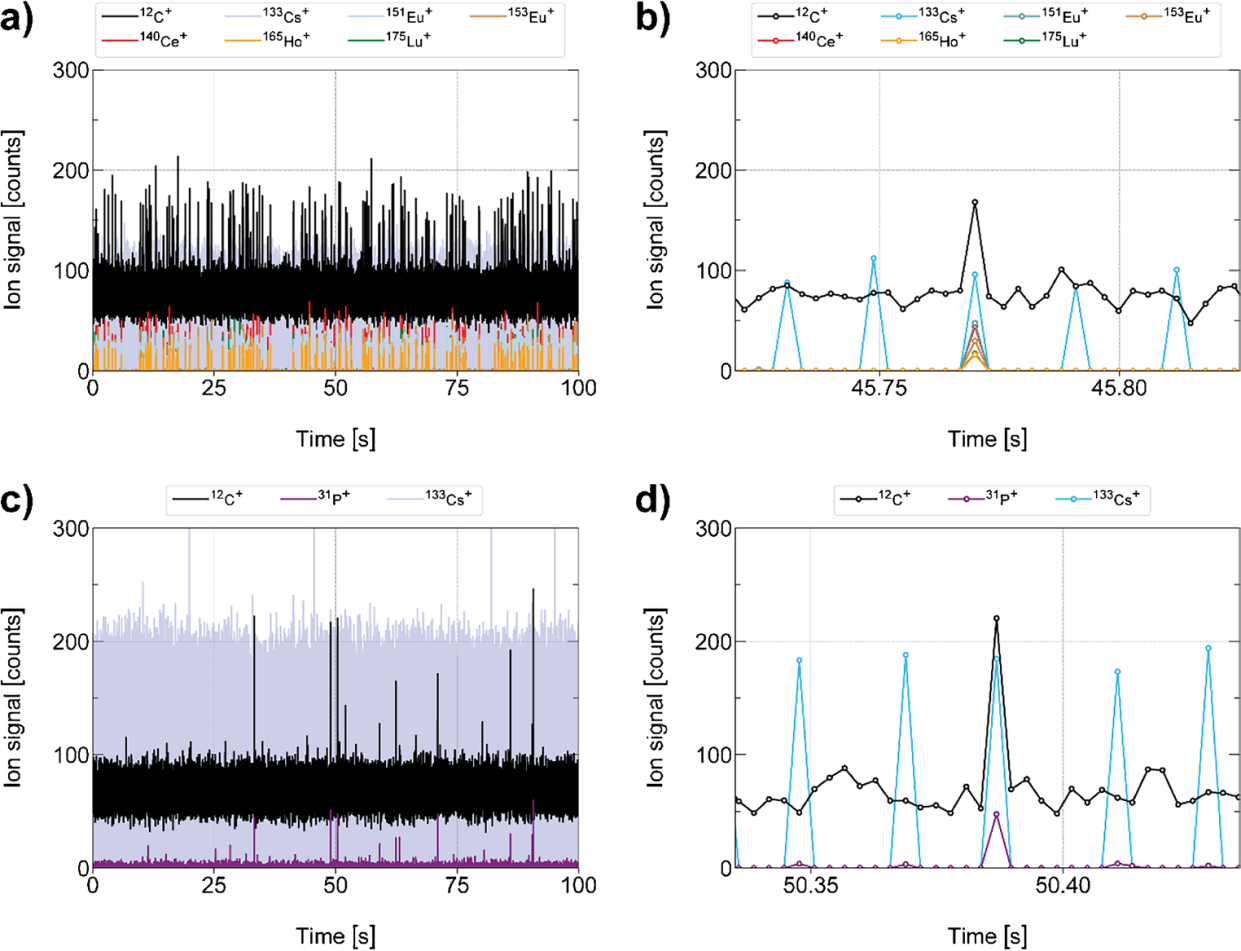
Time-resolved signals recorded for monodisperse droplets carrying beads (**a** and **b**) and cells (**c** and **d**) are shown. Carbon spikes originating from beads and cells were obtained on top of the pronounced background that was found to be stable throughout the acquisition period. Bead events were confirmed according to their spike Ce, Eu, Ho, and Lu and cells according to their P content. **b** illustrates a close-up of subfigure (**a**) showing a typical bead event with REEs and Cs as droplet tracer. **d** illustrates a close-up of subfigure (**c**) showing a typical cell event with P and Cs as droplet tracer

In this study, monodisperse droplets were used to introduce single microbeads (made out of polystyrene) and cells (mouse spleenocytes) into a downward-pointing ICP source. Vertical orientation of the ICP enables quantitative transport of droplets into the plasma independent of the initial droplet size. Analyte elements from the microdroplets were detected via TOFMS and microdroplet-derived signals were registered on the TOFMS time trace by monitoring signal from a droplet tracer element, Cs. For all of the registered microdroplet signals, the signals from ^12^C^+^ and other isotopes of interest, including ^140^Ce, ^153^Eu, ^165^Ho, and ^175^Lu from the REE beads and ^31^P from the cells, were recorded. Sections of the time-resolved ICP-TOFMS data are provided in Fig. 1. As seen, carbon from the plasma produces a stable background of ~ 75 counts/acquisition independent of any dissolved C present in the droplet; at the sensitivity level of the TOFMS, the empty microdroplets (i.e., droplets without beads or cells) do not contain measurable levels of ^12^C. Thus, the detection of any ^12^C^+^ signal spikes above the pronounced ^12^C^+^ background was the result of a bead or cell event. Bead events were recorded and identified based on their spike elements (i.e., Ce, Eu, Ho, and Lu) and cell events were identified based on the coincidence of ^31^P^+^ signal with ^12^C^+^. In Fig. 1a and b, individual bead events, and, in Fig. 1c and d, individual cell events on the ICP-TOFMS time traces are provided to illustrate how bead and cell events can be separated with multielement ICP-TOFMS analysis. Note that exclusively ^12^C^+^ ion signals were considered in this study, as the instrument’s sensitivity was not sufficient to detect ^13^C^+^ ion signals quantitatively. Furthermore, the instrument was tuned specifically towards the low mass region, which limited the sensitivity of elements in the high-mass region (above *m/z* = 80).

The sensitivity for ^12^C^+^ in ICP-MS is low due to the high first ionization potential of 11.26 eV, which results in an estimated 5% ionization in the ICP [27]. Additionally, inherent mass bias of mass analyzer and ion-transmission optics introduce further losses for measurement of low mass-to-charge (*m/z*) species [34]. Here, the ion optics upstream of the TOF mass analyzer were optimized for maximum ion transmission of ^12^C^+^ which led to a sensitivity loss for heavy elements (i.e., ^140^Ce^+^, ^151^Eu^+^, ^153^Eu^+^, ^165^Ho^+^, and ^175^Lu^+^) by a factor of five to ten. The sensitivity for ^31^P^+^ was comparable with our previously reported scICP-TOFMS results [30].

**Fig. 2:**
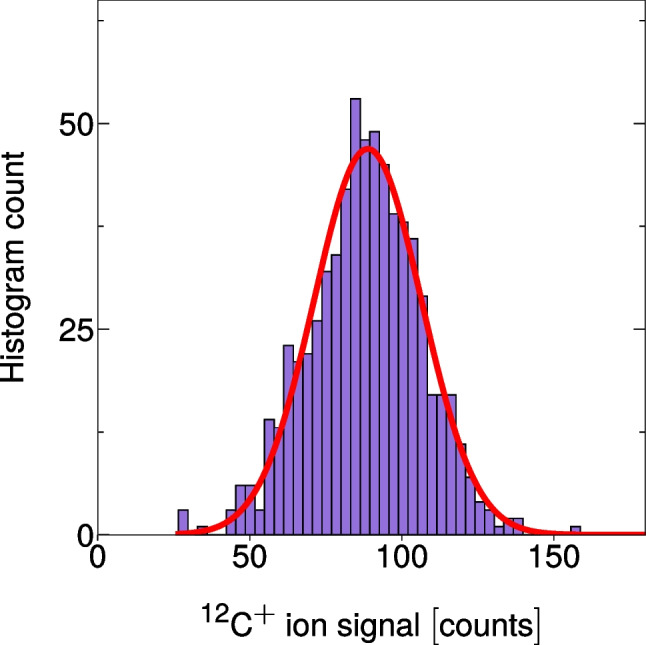
A fitted and background-corrected ^12^C^+^ ion distribution from bead signals is shown

In Fig. 2, we provide an example histogram of the background-subtracted ion signal distribution for the ^12^C^+^ signal distribution for the polystyrene beads introduced via microdroplets. We measured the ^12^C^+^ signal instead of ^13^C^+^ because the higher sensitivity from ^12^C^+^ enabled the detection of all bead events. Bead-derived ^12^C^+^ signals were identified based on concurrent detection of ^140^Ce^+^, ^151^Eu^+^, ^153^Eu^+^, ^165^Ho^+^, and ^175^Lu^+^, which were also in the beads. As the ^12^C^+^ background signal was stable throughout a measurement, the net ^12^C^+^ signal from each bead (or cell) can be determined by subtracting the mean ^12^C^+^ background intensity, which was determined via a Gaussian fit of the background signal histogram. The single-cell dataset was processed in the same manner as the microbead data, with the exception that ^31^P^+^ signals were used as the cell tracer.

**Fig. 3:**
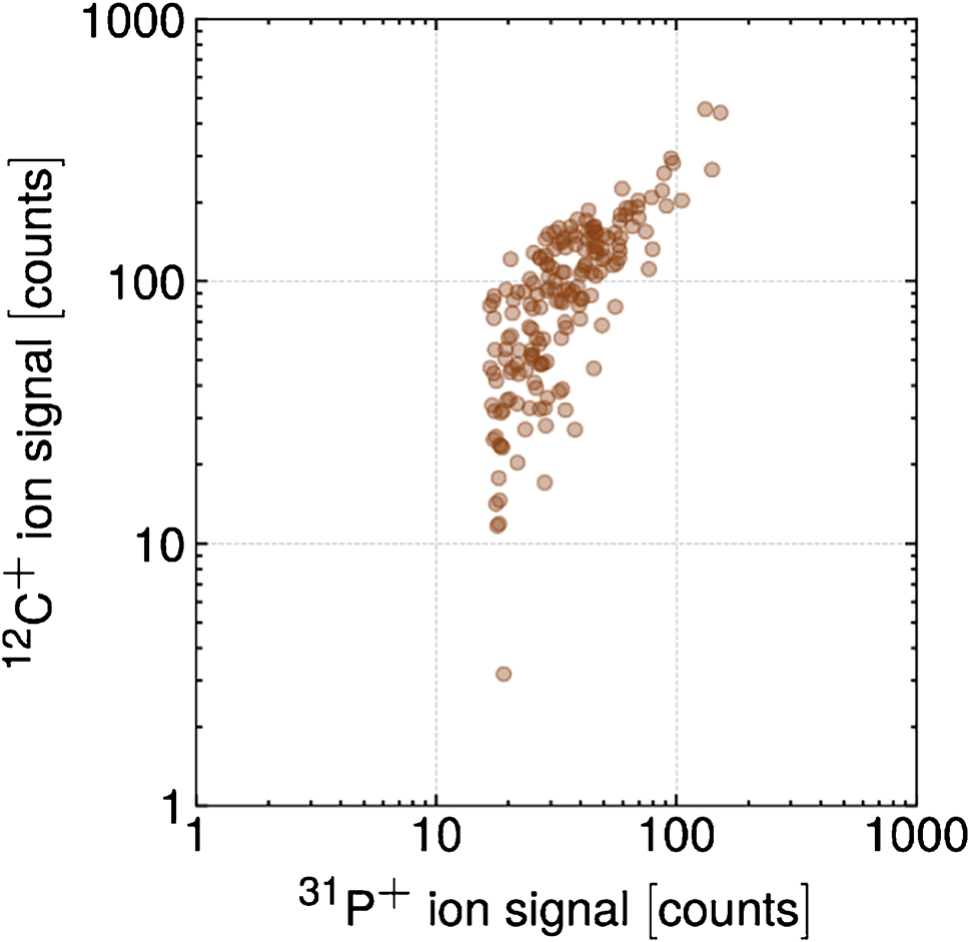
The correlation between ^12^C^+^ and ^31^P^+^ ion signals (background-corrected) that were obtained for single mouse spleenocytes is shown. An increasing P content for cells with increasing carbon content (and vice versa) was observed

In Fig. 3, we plot the correlation of ^12^C^+^ and ^31^P^+^ signals from individual spleenocyte cells. As seen, the recorded ion signals for the cell sample yielded broad distributions spanning one and a half orders of magnitude for ^12^C^+^ and an order of magnitude for ^31^P^+^ signals. This distribution reflects the heterogeneous nature of this spleenocyte sample, which contains different cell types such as B and T cells, monocytes, granulocytes, dendritic cells, and macrophages; all of which show slightly different sizes, and bio-variability [35] ^12^C^+^ and ^31^P^+^ signals appear to be correlated; as cell carbon content increases, so does the amount of P. This correlation might reflect differences in the cell sizes or, more generally, in the biomass. However, it should be noted that cell volume and biomass are not necessarily linearly correlated with each other [2].

As every carbon spike originating from a single-cell event was accompanied by a ^31^P^+^ ion signal, the latter was used to identify and sort out random carbon spikes and signal fluctuations from the distribution of true cell events. The low-count ^31^P^+^ background ion signal distribution did not show a normal distribution, but rather followed a compound Poisson distribution [36]. To identify cell signals, we set a manual threshold for ^31^P^+^ signals one order in magnitude higher than the obtained average background signal.

### Calibration

**Fig. 4:**
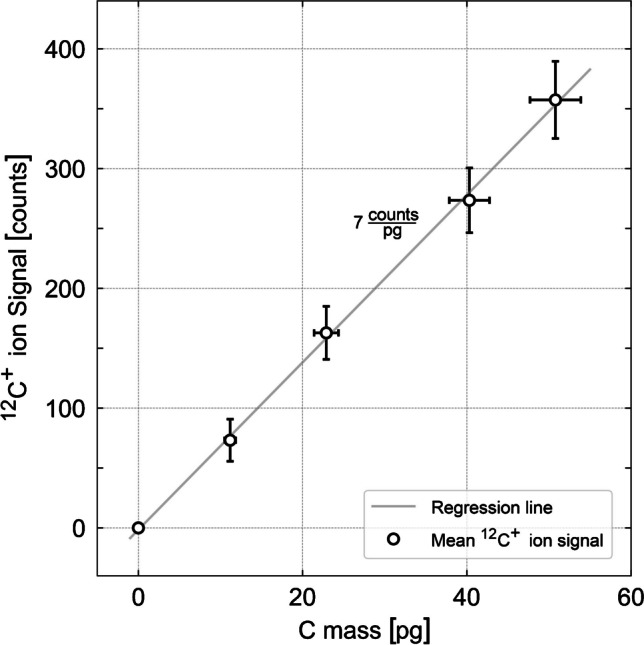
Calibration curve showing the background-corrected ^12^C^+^ ion signal as a function of the carbon mass per sample droplet. A linear regression was carried out yielding *R*^2^ = 0.9988 and the regression line with a slope of approximately 7 counts per pg was plotted to visualize the linear relationship for the calibrated analyte mass range. The uncertainty of the carbon mass was derived from the uncertainty of the droplet size by error propagation

An external calibration was carried out using dissolved glucose in microdroplets as a carbon source [27]. Solutions of four different concentrations of glucose were measured by dispensing thousands of droplets into the plasma so that the detected ion signals from individual droplets yielded a normal distribution for each calibration solution. The mean value was obtained by applying a Gaussian fit to each distribution. Figure 4 shows the background-corrected ^12^C^+^ ion signals as mean values versus the carbon mass per droplet. A linear regression was carried out and the depicted regression line shows a linear correlation between ion signal and mass up to 50 pg C per droplet with *R*^2^ = 0.9988. The limit of detection (LOD) was determined to be 4.83 pg C per droplet.

As demonstrated by Garcia and co-workers, microdroplets form (semi)-dry, solid particles upon the desolvation of the solvent, which can measure up to several micrometers in diameter [33]. According to the glucose concentrations used in this study, semi-dry glucose particles between 7 µm and 13 µm in diameter would have been expected to form serving as suitable calibration proxies for polymer microbeads and cells. However, here the assumption of forming spheres had to be made.

### Quantification and sizing

**Fig. 5:**
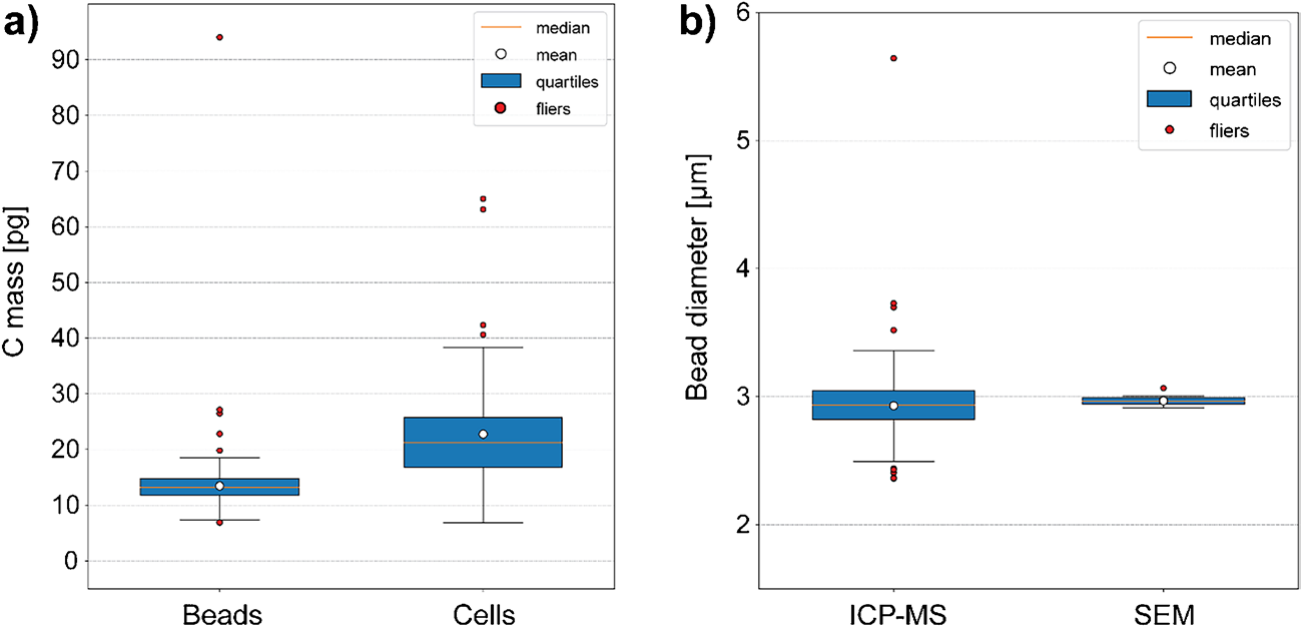
Boxplots showing the carbon mass distribution obtained from single-bead and cell measurements (**a**). Notably, the carbon mass distribution pattern of the beads is much narrower than the distribution pattern of the cells, reflecting the monodispersity of the beads and the heterogeneity of the spleen cell sample. The bead diameter was derived from the determined carbon mass and the size distribution is shown in (**b**). SEM was used to confirm the spherical shape and to measure the diameter of randomly sampled beads

The regression parameters were used to convert the obtained single-bead and single-cell ^12^C^+^ signals into carbon mass per bead and cell, respectively, which is shown in the boxplots in Fig. 5a. Furthermore, the size, i.e., diameter, of the polymer beads investigated was determined and compared with SEM data, which is illustrated in Fig. 5b. A summary of these results is provided in Table 1.

**Table 1:** Summary of sp/scICP-TOFMS and SEM data regarding carbon mass and bead diameter

	*N* total	Mean	STD	Q1	Median	Q3	
Beads	331	13.5	5.2	11.8	13.2	14.8	C mass [pg]
Cells	69	22.8	10.1	16.8	21.2	25.7	
ICP-MS	331	2.93	0.24	2.83	2.94	3.04	Bead diameter [µm]
SEM	24	2.97	0.04	2.95	2.97	3.0	

In the provided boxplots, the corresponding mean values are highlighted by white dots and the median values by orange horizontal lines while interquartiles are indicated by blue boxes with black rectangles. Red dots are referred to as fliers. The ^12^C^+^ ion signals from the beads followed a normal distribution indicated by the shape of the displayed distribution as well as the comparable mean and median values (Fig. 5 and Table 1). The ^12^C^+^ ion signals that originated from cells showed a broad distribution, where the median and mean differed by approximately 1.6 pg, which is consistent with the expected heterogenous distribution (naturally given) of the spleenocytes, in terms of cell types and sizes. Notably, the investigated cells and beads yielded a comparable carbon content. Considering the mean values, approximately 15 pg per cell and 13 pg per bead, respectively, were obtained. Although the beads have a much lower volume in comparison to cells, apparently, comparable absolute amounts of carbon can be expected which underlines the general suitability of beads as calibration standards in mass cytometry.

Assuming the beads were made of 100% polystyrene (*ρ* = 1.05 g cm^−3^) [37] and exist as spheres, the carbon mass distribution shown in Fig. 5a was converted into a bead size distribution and a mean size of the beads was estimated, which is shown in Fig. 5b. A sample of the same bead suspension batch was investigated by SEM and the diameters of 24 randomly selected beads were determined. A very narrow size distribution was obtained which is shown in Fig. 5b. The bead size determined by ICP-MS is in good agreement with the bead size determined by S(T)EM, as will be discussed below.

The number of cell events (*N* = 69) acquired via ICP-TOFMS was lower than for the bead analysis (*N* = 331). A higher number of acquired cell events would have allowed a better statistical description carbon content of the cell population. However, within this proof-of-principle study, we focused on the method development for the analysis of single beads and single cells. We used low cell number concentrations to prevent sampling two individual cells in a single droplet and to reduce the chance of cell agglomeration since cells resuspended in water tend to form agglomerates.

### S(T)EM measurement

**Fig. 6:**
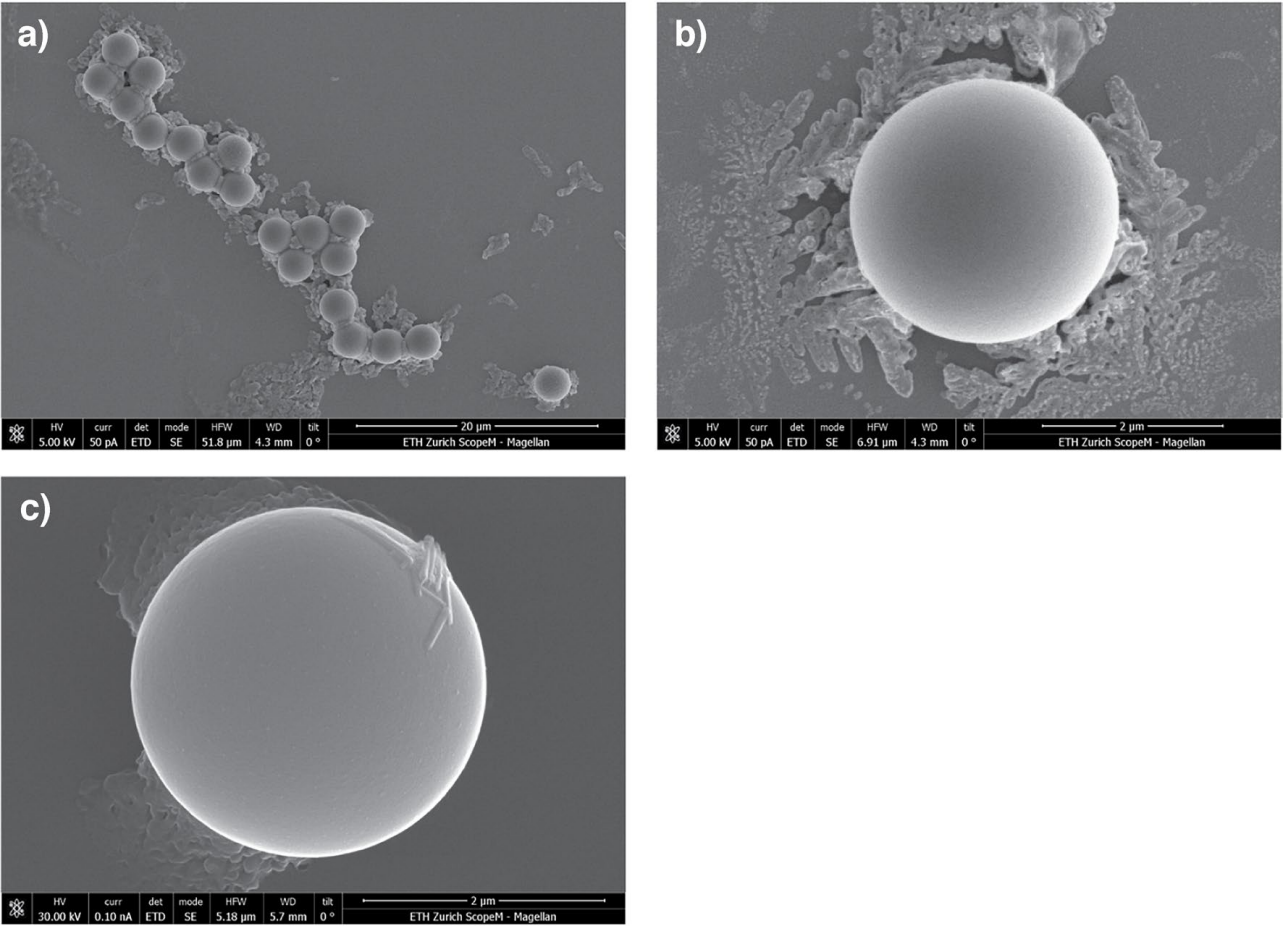
Images of polystyrene beads displaying an array of multiple beads (**a**), and single beads (**b**, **c**) deposited on a Si-wafer which enabled a detailed inspection of the shape and diameter. The beads appeared as spheres surrounded by debris which originated most likely from the suspension medium (buffer/salt) showing a characteristic dendritic growth pattern after drying

We also investigated the bead size, i.e., diameter, and shape by S(T)EM to validate the bead sizing method via spICP-TOFMS. Results are presented in Fig. 6. The array of several beads surrounded by debris can most likely be attributed to residues (buffer/salt) from the suspension medium. (Fig. 6a) Detailed inspection of a bead and the debris is provided in Fig. 6b where a dendritic growth pattern of, e.g., a salt residue, was observed after drying. In Fig. 6c, rod-shaped debris was observed on the bead surface.

A total of 24 beads were investigated by electron microscopy, which is a much lower quantity than the number of beads (331) measured by ICP-MS. The beads showed a spherical shape and appeared to be highly monodisperse with random sampling, which is also reflected by the standard deviation of the SEM data and, additionally, by earlier studies [20, 38, 39]. Thus, given the high uniformity of the beads, the optical analysis of more beads would most likely not have altered the overall statement concluded from the actual dataset, i.e., the agreement of the bead diameters determined by ICP-MS and SEM.

Sizing by quantification of carbon per single bead by ICP-MS with sensitivity calibration via glucose standard microdroplets proved to be a suitable method that allowed a much higher sample throughput than typically observed for S(T)EM. Notably, S(T)EM requires a laborious sample preparation, but provides high-resolution images and thus a remarkable precision. On the other hand, ICP-MS requires almost no sample preparation for polymer beads, but offers significantly lower precision in sizing the particles. This precision is limited by counting statistics and/or droplet-to-droplet signal variations due to axial and radial shifts of the vaporization point of the droplets in the ICP source.

## Conclusion

In this proof-of-concept study, the suitability of the downward ICP-TOFMS prototype instrument for the investigation of individual microparticles and biological cells was demonstrated. The carbon content of single polystyrene microbeads and mouse spleen cells was quantified by ICP-TOFMS using monodisperse microdroplets as particle/cell transporters. Glucose-containing droplets were used for calibration, which enabled the quantification of carbon at single-bead/cell level, while a critical mass of 4.83 pg carbon per droplet was obtained. Importantly, the carbon background was found to be stable throughout the acquisition sequences for the individual samples to enable background correction. A carbon-specific optimization of the ICP-TOFMS’s ion optics was required, which resulted in significantly lower sensitivity for high-mass elements.

As the polystyrene beads were provided as spherical and monodisperse particles, the determined carbon mass per bead was converted into size, resulting in a mean particle diameter of 2.93 ± 0.24 µm. S(T)EM was further used to confirm the beads as spheres and their mean diameter was determined to be 2.97 ± 0.04 µm, which was in agreement with the ICP-MS measurement. Due to the downwards arrangement of the plasma, it can be assumed that even larger micro-materials can be analyzed, e.g., microplastics.

In addition, the carbon content in single mouse spleenocytes was quantified. Compared with the ion signal distribution from microbeads, ion signals from spleen cells yielded a broader distribution, which reflects the heterogenous distribution of the sample in terms of cell types and sizes. Since the cells were resuspended in ultrapure water, a certain fraction of the intra-cellular species such as proteins, lipids, carbohydrates, and nucleic acids were likely washed out due to the osmotic stress which resulted in a biased carbon determination of the individual cells. In the future, the cell sample preparation protocol needs to be further improved to preserve the cellular content as much as possible while also not introducing additional carbon into the sample, for instance, by using a carbon-based fixative [40]. Information on the total carbon mass content of each cell is considered to be very useful when an accurate measurement of the cellular biomass is required as almost the entire dry weight of a cell consists of carbon-based compounds. Depending on the biological variability as well as the instrumental sensitivity and detection limit, the total carbon signal might not be sufficient for the accurate determination of the cellular biomass. Here, an online combination of the downward plasma with flow cytometry might provide the capabilities for further cell characterization.

As plastics and plastic debris have become a major environmental pollution concern, the need for analytical tools and methods that allow for fast throughput and precise characterization and monitoring of nano- and microplastics is steadily increasing [23]. The accurate quantification of carbon in single and sufficiently large micron-sized plastics (e.g., ≤ 10 µm), as demonstrated for 3 µm-sized microbeads in the present study, could therefore be a highly suitable approach for future studies on the analysis of microplastics [24, 25].

### Supplementary Information

Below is the link to the electronic supplementary material.

Supplementary file1 (DOCX 16.8 KB)
