# Supplementary material for: Determination of carbon in microplastics and single cells by total consumption microdroplet ICP-TOFMS
Source: Anal Bioanal Chem. 2023 Dec 8;416(11):2773–81. doi: 10.1007/s00216-023-05064-0 (PMC11009739; doi:10.1007/s00216-023-05064-0)
Supplement: Supplementary file 1 — Supplementary file1 (DOCX 16.8 KB) [file 216_2023_5064_MOESM1_ESM.docx]

**Determination of Carbon in Microplastics and Single Cells by Total Consumption Microdroplet ICP-TOFMS**

Thomas Vonderach, Alexander Gundlach-Graham, Detlef Günther

Table of Contents

[Table S1. Operating conditions for the sample droplet supply system and the downward ICP-TOFMS. 1](#_Toc146975434)

| Operational Parameters | Value |
| --- | --- |
| He flow (upper) | 0.8 L min^-1^ |
| He flow (lower) | 0.1 L min^-1^ |
| Ar flow | 0.4 L min^-1^ |
| Ar auxiliary gas flow | 0.9 L min^-1^ |
| Ar plasma gas flow | 14.2 L min^-1^ |
| ICP power | 1350 W |
| Einzel lens | 7.4 V |
| Sampling depth | 11 mm |
| Injector diameter | 1.5 mm |

# Table S1. Operating conditions for the sample droplet supply system and the downward ICP-TOFMS.
